# Epiploic Appendagitis Mimicking Acute Appendicitis: An Osteopathic Case Report

**DOI:** 10.7759/cureus.32499

**Published:** 2022-12-14

**Authors:** Justin Chin, Basilia Oseguera, Kevin Hon, Christine M Lomiguen, Thomas McBride

**Affiliations:** 1 Family Medicine, LifeLong Medical Care, Richmond, USA; 2 Medical Education, Lake Erie College of Osteopathic Medicine, Erie, USA; 3 Emergency Medicine, New York-Presbyterian Queens Hospital, New York, USA; 4 Family Medicine, LECOM Health Millcreek Community Hospital, Erie, USA; 5 Pathology, Lake Erie College of Osteopathic Medicine, Erie, USA

**Keywords:** appendix, osteopathic structural exam, abdominal pain, epiploic appendagitis, viscerosomatic reflex, chapmans point, osteopathic manipulative medicine, rlq pain, acute appendicitis, primary epiploic appendagitis (pea)

## Abstract

Acute epiploic appendagitis is a rare cause of abdominal pain, often misdiagnosed as acute appendicitis or diverticulitis given similar clinical presentation and findings. The treatment is supportive care and is typically self-limited. The osteopathic structural exam can give insight into pathology and in this case, was suggestive of a non-appendiceal origin of her pain, in which emergent surgery could be avoided. Requiring computerized tomography to identify, acute epiploic appendagitis is a rare cause of abdominal pain and should be considered in the differential diagnosis.

## Introduction

Abdominal pain is a common complaint seen in outpatient primary care and inpatient emergency medicine alike. The differential diagnosis is broad, and it can have benign to life-threatening etiologies, ranging from gastroenteritis to acute surgical abdomen [1]. Depending on the history, physical exam findings, and point-of-care testing/imaging, patients can be managed conservatively or sent to the emergency room for further workup [2]. For right lower quadrant pain, acute appendicitis is a common consideration and often warrants immediate evaluation given the possibility of rupture and the need for surgical intervention. Acute epiploic appendagitis is a less common diagnosis of right lower quadrant pain and is rarely considered. Inaccurate diagnosis often leads to unnecessary hospitalizations, antibiotic therapy, and surgical intervention for patients as it requires computerized tomography (CT) for visualizing and elimination of other etiologies of pain [3,4].

Osteopathic physicians are trained to integrate the medical history of a patient with palpatory examination through the osteopathic structural exam which allows the expansion of differential diagnoses and the consideration of viscerosomatic dysfunction for the localization of symptoms [5]. Here we present a case of acute epiploic appendagitis mimicking acute appendicitis as well as reviewing osteopathic physical exam findings associated with abdominal processes.

## Case presentation

Ms. L is a 54-year-old Latinx female who had presented to the urgent care for four days of diffuse abdominal pain, which had localized to the right lower quadrant earlier that morning. Past medical history was significant for hypertension, hyperlipidemia, and anxiety. Surgical, family, and social history were noncontributory. She denied a prior history of similar abdominal pain, sick contacts, new sexual partners, hematemesis, hematochezia, or changes in urinary/bowel movement. She notes that pain was exacerbated with activity, deep inspiration, and yawning, without any alleviating factors.

Vitals were unremarkable as the patient was afebrile, normotensive, normocardia, and saturating 99% on room air. Physical exam showed decreased bowel sounds on abdominal auscultation, with rebound tenderness and guarding on palpation. Provocative measures such as the McBurney’s point, psoas sign, and obturator test were equivocal. The point-of-care pregnancy test was negative. She was sent to the emergency room due to concern for acute appendicitis, in which abdominal ultrasound was negative. The point-of-care ultrasound was not available and the patient was sent to the emergency room for further evaluation.

**Figure 1:**
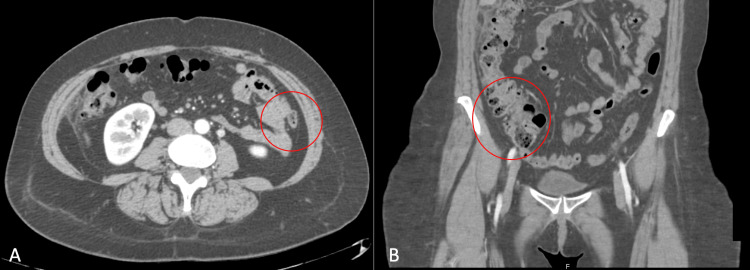
CT scan of abdomen pelvis with and without contrast. A. Transverse view and B. Sagittal view with red circles indicating area of epiploic appendagitis on ascending colon, with surrounding inflammatory fat stranding, and thickening of the adjacent peritoneum. CT: computerized tomography

Upon admission to the emergency department, the patient was given a 30-milligram ketorolac injection for pain as it had significantly worsened in the right lower quadrant. Blood work was unremarkable, with electrolytes within normal limits and mild leukocytosis with a white blood cell count of 11.4 (normal range 4.5-11). CT of the abdomen revealed antimesenteric stranding adjacent to the ascending colon, with central fat attenuation, most compatible with epiploic appendagitis (Figure 1 A-B). No evidence of appendicitis was visualized on imaging. The patient was given two liters of fluids, passed an oral challenge, and was discharged on supportive care and oral ibuprofen.

Outpatient follow-up two days later revealed resolving symptoms and an osteopathic structural exam was performed (Table 1). On exam, the patient continued to have viscerosomatic reflexes in the distribution of the colon and Chapman’s points along the right iliotibial band, which would indicate a colonic etiology. No osteopathic findings were noted that would have been associated with the appendix. Telehealth follow-up in one week revealed a complete resolution of her symptoms.

**Table 1:** Osteopathic structural exam adapted with Chapman's reflexes noted along distribution of ascending colon. T: tissue texture changes A: asymmetry R: restriction of motion T: tenderness

Methods Used for Examination					Severity Scale Key (0 = Background Level/No somatic dysfunction, 1 = Minor TART, 2 = Obvious TART, 3 = TART + key lesions)					
					Region Evaluated	Severity				Somatic Dysfunction and Other Systems
All	T	A	R	T		0	1	2	3	Musculoskeletal and Other Systems
☐	☐	☐	☒	☐	Head and Face	☒	☐	☐	☐	
☐	☐	☐	☒	☐	Neck	☒	☐	☐	☐	
☒	☐	☐	☐	☐	Thoracic levels T1-T4	☒	☐	☐	☐	
☒	☐	☐	☐	☐	Thoracic levels T5-T9	☒	☐	☐	☐	
☒	☐	☐	☐	☐	Thoracic levels T10-T12	☐	☐	☒	☐	Hypertonicity of paraspinals from T10-T11 bilateral
☒	☐	☐	☐	☐	Ribs	☐	☒	☐	☐	Inhalation dysfunction in ribs 5-9 pump, no Chapman in twelfth rib tip
☒	☐	☐	☐	☐	Lumbar	☐	☐	☒	☐	Hypertonic right paraspinals
☐	☐	☐	☒	☐	Sacrum & Pelvis	☒	☐	☐	☐	
☐	☐	☐	☒	☐	Innominate	☒	☐	☐	☐	
☐	☐	☐	☐	☒	Abdomen	☒	☐	☐	☐	Mild tenderness to palpation at right lower quadrant
☐	☐	☒	☐	☐	Right Upper Extremity	☒	☐	☐	☐	
☐	☐	☒	☐	☐	Left Upper Extremity	☐	☒	☐	☐	Slight decrease in internal rotation.
☐	☐	☒	☐	☐	Right Lower Extremity	☐	☐	☒	☐	Chapman’s points along iliotibial band
☐	☐	☒	☐	☐	Left Lower Extremity	☒	☐	☐	☐	

## Discussion

Epiploic appendages are small outpouchings of serosa present on the external surface of the colon that project into the peritoneal cavity [6]. Serving as an easy visual distinction between the small and large intestines, their role in the body is not clearly understood and has been hypothesized to protect the blood supply to the colon during peristalsis or to provide defense/immune response in assisting colonic resorption [7,8]. In turn, epiploic appendagitis is caused by torsion or thrombosis of the central draining vein of the epiploic appendage which can create an ischemic infarction. The true incidence is unknown as epiploic appendagitis is typically self-resolving. In various case series, it is often initially diagnosed as acute diverticulitis versus acute appendicitis (2-7 percent and 0.3-1 percent respectively) [9]. Generally seen in ages 20-50 and four times more common in men, epiploic appendagitis can arise from any segment of the colon, with the rectosigmoid colon being the most common location [10]. Obesity, particularly with increased abdominal circumference, as well as strenuous exercise has been associated with increased incidence, however epiploic appendagitis can also occur in non-obese patients and without known provocation [11].

This case represented a typical case of epiploic appendagitis, however, it also serves as a reminder of the value of the osteopathic structural exam in localizing and aiding in the diagnosis of pathology. Viscerosomatic reflexes, in which visceral and somatic pain afferent nerve signals overlap in the dorsal horn and influence each other, can present as skin erythema for acute processes versus skin blanching for chronic states [12]. Depending on the area of these findings, different associations have been found to correlate between somatic findings and visceral pathology [13-16]. In this case, the appendix is associated with changes at thoracic level 12, which were missing on the structural exam. Similarly, Chapman’s points are “pea-sized gangliform contractions” of musculature that are associated with visceral dysfunction found elsewhere in the body [17]. In the case of colonic pathologies, patients can have Chapman’s points along the iliotibial band that correlate with the area of concern. The case presentation patient had Chapman’s points along the right iliotibial band that corresponds to the ascending colon.

Management is supportive of nonsteroidal anti-inflammatory drugs and is self-limiting as symptoms resolve in 3-14 days [18]. The risk for recurrence has not been well investigated and may be a future area of further research. Reports of inflamed appendages and sequelae associated with adherence to the abominable wall or other viscera have been noted, but are rare [4,19]. Other case reports have also noted increased predisposition to intestinal obstruction, intussusception, and abscess formation after epiploic appendagitis, however, it is unclear if it is coincidental given multiple other comorbidities [3,4,11,19]. Surgical intervention is only done in cases that are refractory to conservative treatment or if there is a worsening clinical picture [20]. Further studies are needed to determine if the osteopathic structural exam is a reliable method in aiding the diagnosis of epiploic appendagitis.

## Conclusions

Epiploic appendagitis is an uncommon cause of right lower quadrant pain and should be considered when developing a differential diagnosis. The clinical course is typically self-limited and resolves with pain medication. The osteopathic structural exam can give insight into ruling out other pathologies such as acute appendicitis, however, imaging modalities such as ultrasound and CT are needed for definitive diagnosis. Greater research is needed in correlating osteopathic findings to physical exam findings and pathology.
